# Health Tracking via Mobile Apps for Depression Self-management: Qualitative Content Analysis of User Reviews

**DOI:** 10.2196/40133

**Published:** 2022-11-23

**Authors:** Ashley Polhemus, Sara Simblett, Erin Dawe-Lane, Gina Gilpin, Benjamin Elliott, Sagar Jilka, Jan Novak, Raluca Ileana Nica, Gergely Temesi, Til Wykes

**Affiliations:** 1 Merck Research Labs Information Technology Merck, Sharpe, & Dohme Zurich Switzerland; 2 Epidemiology Biostatistics and Prevention Institute University of Zurich Zurich Switzerland; 3 Institute of Psychiatry, Psychology and Neuroscience King’s College London London United Kingdom; 4 Division of Mental Health & Wellbeing Warwick Medical School University of Warwick Coventry United Kingdom; 5 RADAR-CNS Patient Advisory Board King's College London London United Kingdom; 6 South London and Maudsley NHS Foundation Trust London United Kingdom

**Keywords:** depression, mental health, health tracking, self-management, data visualization, mobile phone

## Abstract

**Background:**

Tracking and visualizing health data using mobile apps can be an effective self-management strategy for mental health conditions. However, little evidence is available to guide the design of mental health–tracking mechanisms.

**Objective:**

The aim of this study was to analyze the content of user reviews of depression self-management apps to guide the design of data tracking and visualization mechanisms for future apps.

**Methods:**

We systematically reviewed depression self-management apps on Google Play and iOS App stores. English-language reviews of eligible apps published between January 1, 2018, and December 31, 2021, were extracted from the app stores. Reviews that referenced health tracking and data visualization were included in sentiment and qualitative framework analyses.

**Results:**

The search identified 130 unique apps, 26 (20%) of which were eligible for inclusion. We included 783 reviews in the framework analysis, revealing 3 themes. *Impact of app-based mental health tracking* described how apps increased reviewers’ self-awareness and ultimately enabled condition self-management. The theme *designing impactful mental health–tracking apps* described reviewers’ feedback and requests for app features during data reporting, review, and visualization. It also described the desire for customization and contexts that moderated reviewer preference. Finally, *implementing impactful mental health–tracking apps* described considerations for integrating apps into a larger health ecosystem, as well as the influence of paywalls and technical issues on mental health tracking.

**Conclusions:**

App-based mental health tracking supports depression self-management when features align with users’ individual needs and goals. Heterogeneous needs and preferences raise the need for flexibility in app design, posing challenges for app developers. Further research should prioritize the features based on their importance and impact on users.

## Introduction

### Background

Mobile health (mHealth) tools, which often include interventional and health-tracking features [1,2], have been shown to have therapeutic effects on mood and anxiety disorders [3]. These effects can be attributed in part to interventions derived from conventional therapy, such as app-based exercises with cognitive behavioral therapy elements. However, a second complementary effect mechanism has been proposed: by identifying patterns in tracked data, the user learns their own health signals and triggers, enabling proactive health or situation management [4,5]. Such feedback can also facilitate engagement and adherence to mHealth technologies, presenting opportunities for long-term condition management and intervention [6].

To be impactful, these tracking mechanisms must be context sensitive, personally relevant, and readily understandable [7]. This is especially challenging when managing depression, as contextual factors, low mood, past experiences with health tracking, and data literacy affect how individuals interact with or interpret their data [8]. Collaborative design methods, working directly with members of the app’s target audience, are recommended during app development [9]. Although these sessions are often productive and insightful, they are conducted in controlled settings and often reflect hypothetical feedback from a small group of people [9]. Therefore, these studies do not necessarily capture the complex contexts in which apps will be used and instead use brief interactions with a subset of a diverse population to extrapolate preferences for long-term app engagement [10]. Case studies, best practices, and frameworks suggest methodology and general topics (eg, the value of simple visualizations with meaningful data) to explore during these sessions [4,11-13]. However, few externally valid data on patient preferences are available to guide the initial hypotheses and design proposals.

Commercially available mood tracking and health management apps are increasingly used for mental health conditions such as depression, anxiety, and bipolar disorder [14,15]. These apps are gaining popularity as a source of knowledge for app and app feature design, although existing reviews of mental health management apps focus on available features rather than the overall design and experience of the included features from the perspective of the users [1,2,15,16]. User reviews of apps, which are publicly available on app stores, contain valuable insights into the real-world use and user experience of mHealth apps and may provide historical data on app successes and failures, as well as the preferences and experiences of app users [17].

### Aim

The aim of this study was to identify the individual experiences, perspectives, and preferences reported in user reviews of mHealth apps for depression self-management. Through a content analysis of these reviews, we synthesized app reviewers’ self-reported experiences, preferences, and requests to inform the development of future depression health management apps.

## Methods

### Objectives and Research Question

In this study, we explored user experiences of data tracking, visualization, and feedback provided in commercially available mHealth apps for depression self-management. The review protocol was developed a priori, based on the framework proposed by Nicholas et al [17,18].

### Identifying Eligible Apps

Preliminary searches and previous app reviews [16] demonstrated that a comprehensive content analysis of all depression-related app user reviews was impracticable because of the large number of existing apps and the limited search features of app stores. Instead, we identified apps from 3 sources: searches of Google Play and iOS App stores, databases of apps endorsed by health care entities, and “Top App” lists published on the web. First, the first 20 apps [19] were extracted from each app store in July 2020 for each of 5 search terms: “depression,” “depression tracker,” “depression diary,” “mood tracker,” and “mood diary.” All searches were conducted by the same researcher in London, United Kingdom. Each store returned apps ordered by relevance according to the proprietary algorithms of the app stores. These searches yielded 100 apps from each store, many of which were duplicates. We then identified all the apps listed in the National Health Services Apps Library [20] and Orcha [21] using the same search terms. Finally, we identified consumer-oriented reviews on the web, which list the top apps for managing depression. We used a Google search for “Top Depression Apps” published between 2018 and 2020 and extracted all apps listed in the first 5 review articles returned by the search engine’s proprietary algorithm. We designed our search to systematically identify popular apps that were most likely to be identified and used by potential consumers [14]. These sources reflect 3 scenarios through which people with depression are likely to identify health management apps: searches on an app store, endorsement by health care professionals, and endorsement by peers or influencers. To the extent possible, we adopted systematic search best practices, such as establishing search strategies a priori, searching diverse databases, and using multiple search terms [22].

Identified apps were then reviewed for eligibility, as described in Textbox 1.

The eligibility criteria were piloted by 2 reviewers (AP and BE) who underwent a consistency check on 50 apps. Agreement was assessed using Cohen κ [23]. All remaining apps were reviewed for eligibility by a single reviewer (BE) and confirmed by a second reviewer (AP). Disagreements were resolved by discussion.

**App eligibility criteria.**

**Apps were eligible if they:**

- were publicly available either on Google Play or iOS App stores
- were designed for mental health self-management and specifically mentioned depression in the app’s title or description
- included active or passive condition tracking functionality (eg, via a diary function or wearable tracker)
- displayed recorded symptom, health, or wellness data to the user in any textual or graphical format
- were intended for use by individuals living with mental health conditions, rather than professionals or caregivers
- were available in English
- were actively updated and supported, defined as having documentation or software updates within the previous 12 months

### Identifying Eligible User Reviews

In July 2020, user reviews in the English language posted on or after January 1, 2018, were scraped from Google Play and iOS App stores using the Appbot web application (Appbot). This search was updated in January 2022 to investigate longitudinal changes in review content, as several of the included apps were newly released at the time of the original search. Reviews were filtered using keywords (Graph* OR Data* OR Visual* OR Figure* OR Track* OR Info* OR Display* OR Picture*), extracted, and manually screened for eligibility. The user ratings of the app (ie, out of 5 stars) were also extracted. Reviews were eligible if they explicitly or implicitly referred to symptom tracking, use of tracked data, or data visualization. Reviews that discussed the app’s layout or user interface were not eligible. If a review mentioned other topics in addition to tracking or data visualization, only the relevant part of the review was included in the content analysis.

Owing to the large number of available user reviews, we analyzed content to the point of data saturation in a representative sample rather than conducting an exhaustive content review. To prevent sampling bias, we randomized the order of the reviews and extracted the first 50 eligible reviews per app per store (or all eligible reviews when apps had fewer than 50 reviews). The second round of review followed the same procedure as the first, except that we initially extracted a smaller sample size per app (30 reviews per app per store), proportional to the shorter time frame covered by the search.

### Qualitative Analysis

#### Framework Analysis

Overall, 51.09% (633/1239) of the original sample was randomly selected for coding. This subsampling procedure was stratified by app and app store, yielding a maximum of 25 reviews per app per store. We planned to take additional random samples if data saturation (discussed in further sections) was not reached; however, no additional samples were required. In the update, we coded only 150 additional reviews before confirming the themes identified in the original search. Reviews and their metadata were managed and coded using Microsoft Excel.

User review content was explored through framework analysis [24,25] using a coding frame developed in a related systematic literature review [8]. Our protocol allowed for iterative revisions to this frame, including inductive coding, to reflect emerging themes. In all, 3 reviewers coded a set of 100 reviews with deductive codes (ie, those represented in the existing coding frame) and inductive codes derived from the Thomas and Harden [26] inductive approach to data analysis. Each reviewer suggested additions and revisions to the original coding frame. A consensus was reached through discussion, and code definitions were updated and clarified as necessary. Two reviewers (ED-L, GG, or AP) then recoded all user reviews according to the updated coding frame. Coders had the option to propose additional codes during regular review meetings if the frame did not adequately describe the data, but none arose. One reviewer (AP) then reread reviews organized by the code, summarized their content, and proposed themes. Themes were then revised and finalized according to the consensus reached through iterative discussions with the review group.

#### Ensuring Rigor and Establishing Validity

The members of the review team had backgrounds in psychology, epidemiology, digital health technology design, and informatics. We specifically approached this analysis through the lens of a mental health app design, aiming to produce guidance that could guide app developers. Most reviewers had previous experience in qualitative data analysis in the field of digital health and preference research. Those who did not, received training from experienced researchers (AP and SS) on systematic review conduct, framework analysis, and the coding frame before their contributions to the study. The review protocol was drafted a priori and piloted before the start of the study. To the extent possible, review conduct and reporting adhered to the PRISMA (Preferred Reporting Items for Systematic Reviews and Meta-Analyses) guidelines for systematic literature reviews [27]. Whenever a subset of reviews was sampled, the reviews were randomly selected to minimize selection bias. Investigators underwent consistency checks at each stage, for which Cohen κ consistently exceeded 0.7, indicating excellent agreement [23]. All coding was conducted in duplicate to ensure that personal interpretations or human errors did not unduly influence the results. The team held regular discussions, first to clarify aspects of codes or eligibility criteria and then to explore emerging themes.

Sampling adequacy was ensured to the extent possible (given our limited knowledge of the reviewers’ demographics) by monitoring for saturation of the codes. First, we distinguished between the saturation of codes and the saturation of each code’s meaning [28]. We defined the former as confirmation of the code’s presence in the data set and the latter as the degree to which codes or themes are exemplified in the data set [29]. To evaluate the saturation of the codes, we used the Fugard and Potts [30] method to predict saturation based on probability theory. This approach was appropriate for our data set, given our large, random sample of reviews and our predominantly deductive approach to data analysis [30,31]. Our data set provided >80% power to identify 5 instances of themes mentioned by 1% of the population. We chose a cutoff of 1% to reflect the shallow nature of this data set, assuming that not all who experienced a code would describe it in their review, and 5 instances because this was typically the number of observations required to achieve repetition of content within the codes. Therefore, we confirmed the code to be present in the data set when we observed 5 instances of the code. We further ensured that saturated codes were present in the reviews of more than one app to reduce spurious or app-specific findings. To ensure validity and saturation of meaning, we qualitatively monitored coded reviews for (1) congruence with the meaning established in the original coding frame, (2) new meaning or content that did not arise in the original coding frame, (3) repetition of meaning and content within each code, and (4) repetition of the original search and update. We then conducted quantitative analyses and member checking with our patient advisory board to assess the influence of potential confounders and ensure the face validity of our results.

### Quantitative Analysis

We calculated the sentiment of each coded review using the polarity score generated by the Python programing language’s TextBlob library [32]. Sentiment analysis describes the affective or emotional tone presented in the text [33] based on psychological evidence of the emotional meaning of constituent words or phrases [34,35]. It has been used in several health-related cases, such as in detecting language associated with depressive symptoms [36,37], extracting opinions on health care–related topics [38], and identifying mental health stigma in social media data [39]. The score derived from this analysis identifies text with positive, neutral, or negative tones on a continuous scale, where scores closer to −1 are very negative, scores closer to +1 are very positive, and a score of 0 is neutral.

The normality of sentiment scores and user ratings was assessed visually and via the Shapiro-Wilk test [40]. Ratings and scores were not normally distributed; therefore, nonparametric statistics were used to identify differences among app stores, apps, and codes. Kruskal-Wallis tests [41,42] and Wilcoxon signed rank tests [43] identified differences in sentiment scores and ratings among subgroups and over time. Cumulative link mixed models [44] examined differences in ratings among subgroups, whereas linear mixed models [45] examined differences in sentiment scores. Random effects of individual apps were assumed for both methods, and the effects were assessed for statistical significance using likelihood ratio tests [44,45]. Fisher exact tests assessed yearly changes in code frequency [46,47]. The significance level was assumed to be α=.05, and all analyses were corrected for multiple testing using the Benjamini-Hochberg procedure [48]. All statistical analyses were conducted in R (version 4.1.0; R Foundation for Statistical Computing) using the RStudio environment (version 1.4.1717) [49].

### Patient and Public Involvement

This review is part of a study series that was codeveloped with the members of our patient advisory board. The board was involved in designing the study, developing search terms, reviewing the analysis plan, member checking the coding frame, and interpreting the results. A representative (RIN), who is one of the authors of this manuscript, critically reviewed the manuscript.

### Ethical Considerations

Ethics approval was not required as we used publicly available, nonsensitive data, that was anonymized.

## Results

### Included Apps and User Reviews

The searches identified 130 unique apps, of which 26 were eligible for inclusion. An app selection flow diagram similar to that recommended for systematic literature review reporting [27] is provided in the Figure S1 in Multimedia Appendix 1, and the characteristics of the included apps are provided in Table S1 in Multimedia Appendix 2. In the first round of analysis, we extracted 1239 eligible user reviews from these apps. All eligible reviews were included in the sentiment analysis, and 633 were included in the framework analysis. In the update, we extracted 702 eligible user reviews, of which 150 (21.4%) were included in the framework analysis before saturation was reached. The 1941 eligible reviews generally had positive sentiment scores (median 0.27, IQR 0.14-0.40, range −0.70 to 1.00) and most accompanied positive user ratings (median 5, IQR 4-5, range 1-5). Ratings and sentiment scores differed among apps (*P*<.001). Ratings did not differ among app stores (*P*=.84), but sentiment scores were slightly lower in iOS App store than in the Google Play store (median for Google Play store 0.30, IQR 0.16-0.44; for iOS 0.24, IQR 0.12-0.36; *P*<.001) after adjustment for app-related random effects. Ratings and sentiment scores of individual apps decreased over the 4 years of the review period, both overall (ratings *P*<.001; sentiment scores *P*=.009) and independently for several apps (Table S2 in Multimedia Appendix 2).

Three themes emerged from the framework analysis of 783 reviews: “Impact of app-based mental health tracking,” “Designing impactful mental health–tracking apps,” and “Implementing impactful mental health–tracking apps.”

### Impact of App-Based Mental Health Tracking

Users described how tracking their health through apps provided *structure and organization* for their health management, improved their ability to *recall past experiences*, and increased their *self-awareness*, allowing them to identify patterns and track their progress. This enabled them to use interventions, self-care, or preventive actions to *proactively self-manage* their depression and *reduce their symptoms*. Experiencing these impacts affected the reviewers’ willingness to *engage with the app* regularly. Illustrative quotes are provided in Table 1. Reviews reporting the impact of health tracking were accompanied by higher ratings, although the sentiment scores of these reviews did not differ from those of the entire corpus (Table S1 in Multimedia Appendix 1).

**Table 1:** Themes, underlying codes, and illustrative quotes comprising the theme “Impact of app-based health tracking.”

Codes^a^	Code definition	Illustrative quotes
Increase self-awareness; N=193; deductive	Any description of how visualizations related to or affected service users’ self-awareness, usually regarding symptoms and triggers. Subcodes describe the use of visualizations to identify patterns (eg, identify responses to a trigger, relating specific activities to symptoms) or seeing progress (eg, seeing change over time or in response to an intervention)	“Very helpful for tracking your mood and helping you feel better. It takes you into your thoughts to realize why you’re feeling how you do and to help you cope. It is very organized in a helpful way with a simple graph...” [Youper, 2021, 4 stars]
Provide structure and organization; N=188; deductive	Any description of how tracking affected (actually or hypothetically; implicit or explicit) service users’ ability to organize or structure their memories, symptom data, or approach to self-management	“I use it to track my energy and attention levels to create a more productive daily schedule.” [iMood, 2021, 5 stars]
Enable proactive self-management; N=97; deductive	Any description of how visualizations affect (actually or hypothetically; implicit or explicit) participants’ ability or motivation to self-manage their conditions	“I’m loving this app! It has so many features to explore that help me grow and learn. The training is spot on, and I love the ability to keep track of my emotions in such detail. The tracker has helped me spot areas that I can focus on to keep me in a healthy state of mind. Highly recommend!” [Lift, 2019, 5 stars]
Alter symptoms; N=68; inductive	Any discussion of how visualizing data directly or indirectly changed an individual’s symptoms or an individual’s perception of their symptoms	“This app is wonderful. The design is playful and fun with the cloud mascot and the ability to earn stickers and the other unobtrusive progress tracker. More importantly, it works. I have recently been under a lot of stress. This app has made me feel much more grounded and myself than I have felt in a long time.” [MyLife Meditation, 2018, 5 stars]
Enable engagement with apps; N=66; deductive	Any description of how tracking affected (actually or hypothetically; implicit or explicit) engagement with remote monitoring technologies, either within a single session of using the app or over time	“Incredible app for free. I used to really dislike mood trackers and always ended up removing them, but this is brilliant. Lovely to use, lots of easy settings and so many areas to track. Will be using this for a long time.” [Bearable, 2020, 5 stars]
Affect self-image; N=21; deductive	Any description of how visualizations affect (actually or hypothetically; implicit or explicit) service users’ perception of themselves, their illness, or their abilities, either positively or negatively	“I’m a sensitive person, so many things ‘set me off’ in a different mood. Aside from seeing a therapist regularly, this app has made a huge difference in how I view myself, my thoughts, and my emotions.” [Moodpath, 2020, 5 stars]
Improve recall of past experiences; N=9; deductive	Any description of how visualizations affect (actually or hypothetically; implicit or explicit) service users’ ability to remember or recount historical symptoms or experiences	“...My favorite feature is the mood tracker which lets you track your mood throughout the day and then averages it. You also can write a little explanation about your mood—which if you’re like me with not the best memory it’s so nice to be able to go back and see those entries. It also helps me realize that setbacks I face throughout my day [and would ordinarily obsess about] are just little blips. I can see that despite my panic attack the day is still good, it hasn’t been completely ruined. It’s been very helpful for me to have something visualizes that so well...” [Bearable, 2020, 5 stars]
Validate current experiences; N=7; deductive	Any description of how tracking affected services users’ perception of the validity, acceptability, normality, or realness of their own symptoms	“It is really helpful to track my mood. It helps me pause and reflect. It’s easier to challenge my thought in private and accept reality.” [Woebot, 2019, 5 stars]

^a^The number of times each code was identified (N) and whether the code was deductive or inductive.

### Designing Impactful Mental Health–Tracking Apps

#### Overview

Reviewers frequently attributed their ability to achieve (or not achieve) the desired impacts of mental health tracking to aspects of an app’s features and designs. Although a single set of codes was relevant throughout this theme, the review content related to app features and design preferences was grouped into two stages of health tracking: (1) recording data and (2) reviewing and visualizing data. Two additional subthemes, “customization” and “preference moderators, appeared across multiple aspects of app design. Illustrative quotes are provided in Table S2 in Multimedia Appendix 1.

#### Recording Data

The reviewers discussed a variety of *formats* for recording data, including scales, selection of prepopulated options, free text, pictures, emojis, and dialogue with chatbots. They described how, through any mechanism, data entry must be *simple*, despite the *complexity* of the data that they often need to track. For them, simplicity meant that data reporting should be quick, easy, and readily accessible, especially during low moods when they have reduced motivation to track their symptoms. However, oversimplifying apps by reducing the number of *categories available* to track often undermined their usefulness. Scaled options such as mild, moderate, and severe or simple emotions such as sadness or happiness were often perceived as too vague to be meaningful. Tracking moods through emojis evoked opposing responses; some reviewers found them too generic to be meaningful, whereas others appreciated their simplicity. For some reviewers, reporting data through dialogue, such as through a chatbot, was perceived as more natural and private than through a journal or questionnaire, making them more willing to document their experiences.

User reviews described how individuals have unique symptoms, triggers, and environments; therefore, individual tracking needs extend beyond mood and emotions. Preferences related to tracking mechanisms were often *moderated* by *context* and past *experiences with health tracking*. *Annotation* with *contextual* information was often requested to aid future data interpretation. This included the date, day of the week, and time of the symptom, as well as noteworthy events that happened during the day. This was most frequently described or requested as a free-text field that could be accessed when reviewing the data. Reviewers also liked using pictures and tags to contextualize their data.

Reviews consistently praised or requested the ability to *customize* the data, mood, and symptoms tracked in the app. Suggestions included sleep, daytime naps, diet, water and coffee intake, exercise, weight, menstruation, medications, stressful events or conversations, and use and effectiveness of coping strategies. Conversely, users described how tracking could be overwhelming if a data-reporting mechanism provided too many options. Similarly, the required *time frame* or frequency of data reporting differed from person to person. Frequently, apps only allowed users to log 1 mood or diary entry per day, although the ability to log multiple times per day was sometimes available as a paid feature. Once-daily tracking was generally considered insufficient to track patterns, triggers, and health status, as emotions and symptoms evolve throughout the day.

#### Reviewing or Visualizing Data

Reviewers described color coding, statistical summaries, graphs and calendar views, and nontraditional visualizations, such as word clouds, as valuable and engaging *formats*. They also suggested that it is important to visualize and *compare multiple data streams* when attempting to identify patterns. However, relevant data streams differed between individuals and *contexts*, and many noted that it was important to *customize* which variables to visualize and compare. Additional contextual or clinical information was also frequently requested to aid interpretation visualization. However, several reviews have cautioned against making graphs overwhelming, suggesting that the balance between *simplicity and complexity* must be carefully considered during design.

They also suggested that the *time frame* represented in the visualizations should be flexible or customizable because visualizations over different time frames were useful in different contexts. Shorter time frames helped individuals reflect on their days and identify triggers, especially during periods of low mood. Visualizations covering longer time frames helped individuals see progress or trends and were useful as communication tools for physicians.

### Implementing Impactful Health-Tracking Apps

#### Overview

Reviewers also discussed aspects of app implementation that affected their health-tracking practices and abilities. This theme comprised 3 subthemes: “integrating app-based tracking into a larger health ecosystem,” “costs, finance, and paywalls,” and “technical issues.” Illustrative quotes are provided in Table S3 in Multimedia Appendix 1.

#### Integrating App-Based Tracking Into a Larger Health Ecosystem

This theme is related to *communication and sharing*, *generating reports and exporting data*, *connectivity, and interoperability*. Reviewers frequently described or requested the ability to export their data and generate reports, either for personal use or to facilitate communication with others. Storing data in the app alone was often considered insufficient, and reviewers frequently described their desire to export their data. They conducted additional analyses outside the app and archived the data to prevent data loss. Often, reports and visualizations were used to communicate with health care providers during therapy sessions. When data entry required an internet connection, reviewers requested offline modes to enable regular and reliable tracking regardless of the environment and context. They also regularly praised or requested integration with other health apps and appreciated when apps could track all necessary data in one place (symptoms, mood, medication, diet, etc); therefore, duplicate input was not necessary.

#### Costs, Finance, and Paywalls

*Cost, finance, and paywalls* were usually discussed in terms of whether the app or premium version was worth purchasing, although insufficient detail was provided to establish which factors made the apps worth purchasing. Originally, apps were either free with advertising, one-time purchase, or “freemium” with free features but the option for a paid upgrade. These options were generally well received by reviewers who weighed the pros and cons of paying to track their health data. However, several apps have changed to a subscription model in 2020 or 2021, with many or most tracking features requiring monthly or weekly fees. Many reviewers considered this model overpriced, unaffordable, or exploitative and often reported switching to other tracking apps for this reason. Reviewers also discussed the effects of data loss when apps updated or changed their access models. The included ratings associated with these apps decreased significantly following these changes (Multimedia Appendix 2). This change also preceded the changes in the frequency of several codes over time (Table S2 in Multimedia Appendix 2), reflecting the reduced access and customizability of features that were affected by a paywall.

#### Technical Issues

The most common technical issues were data loss and inaccuracies in the app data. Data loss was frequently devastating, as apps held years of insight and a wealth of knowledge reviewers used for self-management. Other issues included dates and times displaying inaccurately in visualizations and issues in exporting data when export was supposed to be possible. Reviews reporting technical issues received significantly lower ratings and sentiment scores, and the proportion of reviews reporting issues increased over time (Table S1 in Multimedia Appendix 1).

## Discussion

### Principal Findings

This review considers spontaneous user feedback on publicly available apps, reflecting real-world experiences with app-based mental health tracking. Reviews tended to be positive and suggested that simple user experiences, customizability, interconnectivity, and sophisticated data visualizations are desirable and impactful features of health tracking. These findings validate and elaborate on a systematic review of user feedback in academic studies [8]. Similar to the feedback generated in research settings, user reviews described how individuals with depression used app-based health tracking to identify trends, track progress, and communicate with their therapists. User reviews have also emphasized the need for apps to be customizable and context sensitive. The similarities among these findings are encouraging, suggesting that previous laboratory-based studies on apps for mental health management [50-56], which were largely hypothetical or limited in time frame, yielded externally valid themes. This analysis of user reviews based on these findings provides additional details, practical insights, and specific design considerations that have not been discussed in academic publications.

### Design Considerations

The review content provided additional details that were not described in peer-reviewed studies, which may be useful when designing and implementing mental health–tracking features for mobile apps (Textbox 2).

**Design considerations for mental health–tracking apps.**

**Designing impactful health-tracking mechanisms**

- Impactful designs should allow app users to...
  - track data which is relevant to their own, highly individual experiences
  - track multiple data streams, multiple times per day
  - capture the context of the experiences or scores they reported
  - review data over different time frames for different purposes
  - strike a balance between ease of tracking and precision of the data; for example, by supplementing default responses with options for additional detail
  - capture and communicate health insights at a level which is appropriate for an individual’s health and digital literacy

**Implementing impactful health-tracking mechanisms**

- When planning for launch and implementation, app designers should consider the following:
  - enable apps to be used in conjunction with other technical and nontechnical health resources
  - minimize the potential for data loss through local and cloud storage, offline modes, backups, enabling manual downloads, and archiving
  - ensure that apps work accurately across time zones
  - address technical issues in a timely manner to mitigate impacts on data access and accuracy
  - consider impacts to current users—especially with regard to data access—before upgrades and business model changes

First, reviews indicate that the content and granularity of tracked data should be relevant to the individual user’s conditions, needs, goals, and experiences, which may change across contexts and over time. Many reviewers needed to record and visualize multiple types of data simultaneously, multiple times per day. However, the types of data that app users wished to track varied from person to person, as did the relevant time frames over which users wished to review their data. App reviews also suggested conflicting preferences between the ease with which data are recorded and the detail or precision with which data can be captured. Some apps’ data-reporting mechanisms were described as simple yet too generic to be useful, others were highly detailed but too cumbersome to complete regularly. This tension made it more difficult to address disparities in health and digital literacy across the population [57]. App-based health-tracking mechanisms must capture and convey health information at a level that matches the needs and competencies of a diverse intended audience [58].

Our findings imply a need for flexibility and choice in the level of detail captured and conveyed during mental health tracking. However, apps should strive to avoid common pitfalls of health communication, in which health information is presented in ways that are too generic, technical, complex, abstract, or didactic for users to interpret readily [59]. Apps should provide flexibility in ways that maximize informational value minimizing the cognitive effort involved in data entry and interpretation [13]. App reviews suggested several ways to achieve this balance. Responsive recommendations when tracking emotions, such as suggesting nuanced synonyms based on an initial entry, may allow users to explore and capture detailed data quickly without having to search through long lists. Searching, scrolling, and zooming functions on visualizations may allow users to view data, and therefore patterns, over time frames that are personally relevant. Finally, options to “dig deeper” into visualized data, for example, by clicking on a data point to reveal additional details, analysis, and contextual information, may be beneficial to users who require more detail without overwhelming those who would struggle to interpret it.

Reviews have also demonstrated that the contextual diversity of an app’s target audience leads to additional technical and implementation challenges. App users described having multiple technical and nontechnical health resources at their disposal (eg, other apps, wearables, caregivers, and health care professionals); therefore, mental health–tracking apps should be compatible with these resources when possible. It is important for app reviewers to report data at convenient times soon after the occurrence of meaningful events. Connectivity issues, such as intermittent internet access, sometimes prevented timely data input, and offline modes were requested in reviews. Many reviewers have reported data loss owing to technical issues or app upgrades. Designers should consider options to prevent data loss, such as cloud storage, regular backups, or manual downloads and archiving. Finally, the reviewers reported several instances in which the app updates and changes to an app’s business model affected their health-tracking practices. Several apps have changed their feature offerings and business models over the 4 years covered by this review, adding web-based communities, digital cognitive behavioral therapy packages, and remote therapy platforms. This pivot and subsequent expansion of paywalls made tracking unaffordable for many reviewers and caused users to lose access to longitudinal data. App providers should be conscious of the ethical implications of their product development and business decisions, particularly when these decisions may affect data access [60], as changes to app features or payment plans could adversely affect users who have integrated the app into their long-term health management strategies.

### Strengths, Limitations, and Future Work

Unlike previous studies on data visualization preferences, this study analyzed spontaneous, user-generated data to understand real-world perspectives, experiences, and challenges with depression self-management apps. This approach has the potential to produce insights with greater external validity than those obtained in laboratory settings. However, this method also has several limitations. An advanced, reproducible search method does not exist for Google search engines or app stores; therefore, this review did not include all available depression management apps. It is plausible that the location and search history of the reviewers who conducted these searches may have influenced which apps were identified and included in this review. This review also inadvertently included user reviews both before and during the COVID-19 pandemic, which had strong adverse effects on global mental health [61-63]. Digital interventions have been widely recommended for the population during this time [64]. All included apps were released before the pandemic, and we opted not to expand the pool of included apps in our updated search, in part, to mitigate the pandemic’s confounding effects on app design. However, the pandemic may have influenced app design and review content.

The use of app reviews has also resulted in a relatively poorly characterized source population compared with purposively selected participants in academic research. Previous studies have described how experience with remote monitoring technology health status, cultural context, health and digital literacy, and other factors moderate user preferences for visualization designs [8,11]. It is important to consider the data through this lens to understand the potential sources of bias and generalizability of our findings.

Many reviews explicitly compared an app to past experiences, in which another app did not meet the reviewer’s needs. However, reviews of the included apps were generally positive, suggesting that users less frequently provided negative reviews when an app did not meet their needs. As a result, the content reviewed here may reflect a bias toward positive experiences. In addition, the duration of app use was unclear in most reviews. Future work should explore the features that yield positive first impressions and those associated with long-term app adherence.

It is also impossible to directly assess the health, digital, or data literacy of the reviewers. However, to generate the included content, users must have sufficient literacy to identify, download, use, and review health apps on a smartphone. Therefore, we presume that digital and health literacy in this population is moderate to high. Many reviewers requested sophisticated reports and visualizations or wished to export and analyze their data independently. This exceeds the expected data literacy of the general population [65], indicating a selection bias. Therefore, the results should be interpreted with caution in populations with low health, digital, and data literacy.

### Conclusions

Data visualizations support depression self-management when they align with the users’ individual needs and goals. To achieve this alignment, personalized data entry mechanisms and visualization content are often desired or necessary. These heterogeneous preferences pose a challenge for app developers, and further research should prioritize features based on their importance and impact on service users. Despite the limitations of the review-based content analysis, it contains readily attainable, free, and externally valid insights that complement formal qualitative research.

## Appendix

Multimedia Appendix 1 Supplementary figures and tables describing the PRISMA (Preferred Reporting Items for Systematic Reviews and Meta-Analyses) flow diagram, coding frame, and illustrative quotes.

Multimedia Appendix 2 Supplementary tables describing the characteristics of included apps and their reviews.

## Abbreviations

mHealth: mobile health
PRISMA: Preferred Reporting Items for Systematic Reviews and Meta-Analyses

## References

[1] Qu C, Sas C, Daudén Roquet C, Doherty G (2020). Functionality of top-rated mobile apps for depression: systematic search and evaluation. JMIR Ment Health.

[2] Myers A, Chesebrough L, Hu R, Turchioe MR, Pathak J, Creber RM (2020). Evaluating commercially available mobile apps for depression self-management. AMIA Annu Symp Proc.

[3] Weisel KK, Fuhrmann LM, Berking M, Baumeister H, Cuijpers P, Ebert DD (2019). Standalone smartphone apps for mental health-a systematic review and meta-analysis. NPJ Digit Med.

[4] Almalki M, Gray K, Sanchez FM (2015). The use of self-quantification systems for personal health information: big data management activities and prospects. Health Inf Sci Syst.

[5] Rickard N, Arjmand HA, Bakker D, Seabrook E (2016). Development of a mobile phone app to support self-monitoring of emotional well-being: a mental health digital innovation. JMIR Ment Health.

[6] Simblett S, Matcham F, Siddi S, Bulgari V, Barattieri di San Pietro C, Hortas López J, Ferrão J, Polhemus A, Haro JM, de Girolamo G, Gamble P, Eriksson H, Hotopf M, Wykes T, RADAR-CNS Consortium (2019). Barriers to and facilitators of engagement with mHealth technology for remote measurement and management of depression: qualitative analysis. JMIR Mhealth Uhealth.

[7] Marzano L, Bardill A, Fields B, Herd K, Veale D, Grey N, Moran P (2015). The application of mHealth to mental health: opportunities and challenges. Lancet Psychiatry.

[8] Polhemus A, Novak J, Majid S, Simblett S, Morris D, Bruce S, Burke P, Dockendorf MF, Temesi G, Wykes T (2022). Data visualization for chronic neurological and mental health condition self-management: systematic review of user perspectives. JMIR Ment Health.

[9] Altman M, Huang TT, Breland JY (2018). Design thinking in health care. Prev Chronic Dis.

[10] Henze N, Pielot M (2013). App stores: external validity for mobile HCI. Interactions.

[11] Arcia A, Merrill JA, Bakken S, Edmunds M, Hass C, Holve E (2019). Consumer engagement and empowerment through visualization of consumer-generated health data. Consumer Informatics and Digital Health: Solutions for Health and Health Care.

[12] Wu DT, Chen AT, Manning JD, Levy-Fix G, Backonja U, Borland D, Caban JJ, Dowding DW, Hochheiser H, Kagan V, Kandaswamy S, Kumar M, Nunez A, Pan E, Gotz D (2019). Evaluating visual analytics for health informatics applications: a systematic review from the American Medical Informatics Association Visual Analytics Working Group Task Force on Evaluation. J Am Med Inform Assoc.

[13] Khasnabish S, Burns Z, Couch M, Mullin M, Newmark R, Dykes PC (2020). Best practices for data visualization: creating and evaluating a report for an evidence-based fall prevention program. J Am Med Inform Assoc.

[14] Wasil AR, Palermo EH, Lorenzo-Luaces LL, DeRubeis RJ (2022). Is there an app for that? A review of popular apps for depression, anxiety, and well-being. Cogn Behav Pract.

[15] Wasil AR, Venturo-Conerly KE, Shingleton RM, Weisz JR (2019). A review of popular smartphone apps for depression and anxiety: assessing the inclusion of evidence-based content. Behav Res Ther.

[16] Shen N, Levitan MJ, Johnson A, Bender JL, Hamilton-Page M, Jadad AA, Wiljer D (2015). Finding a depression app: a review and content analysis of the depression app marketplace. JMIR Mhealth Uhealth.

[17] Nicholas J, Fogarty AS, Boydell K, Christensen H (2017). The reviews are in: a qualitative content analysis of consumer perspectives on apps for bipolar disorder. J Med Internet Res.

[18] Nicholas J, Larsen ME, Proudfoot J, Christensen H (2015). Mobile apps for bipolar disorder: a systematic review of features and content quality. J Med Internet Res.

[19] Rossi MG, Bigi S (2017). mHealth for diabetes support: a systematic review of apps available on the Italian market. Mhealth.

[20] NHS Apps Library. NHS Digital.

[21] ORCHA.

[22] (2009). Systematic Reviews: CRD's guidance for undertaking reviews in health care. Centre for Reviews and Dissemination.

[23] Cohen J (1968). Weighted kappa: nominal scale agreement with provision for scaled disagreement or partial credit. Psychol Bull.

[24] Ritchie J, Lewis J, McNaughton Nicholls C, Ormston R (2013). Qualitative Research Practice: A Guide for Social Science Students and Researchers. 2nd edition.

[25] Gale NK, Heath G, Cameron E, Rashid S, Redwood S (2013). Using the framework method for the analysis of qualitative data in multi-disciplinary health research. BMC Med Res Methodol.

[26] Thomas J, Harden A (2008). Methods for the thematic synthesis of qualitative research in systematic reviews. BMC Med Res Methodol.

[27] Tricco AC, Lillie E, Zarin W, O'Brien KK, Colquhoun H, Levac D, Moher D, Peters MD, Horsley T, Weeks L, Hempel S, Akl EA, Chang C, McGowan J, Stewart L, Hartling L, Aldcroft A, Wilson MG, Garritty C, Lewin S, Godfrey CM, Macdonald MT, Langlois EV, Soares-Weiser K, Moriarty J, Clifford T, Tunçalp Ö, Straus SE (2018). PRISMA extension for scoping reviews (PRISMA-ScR): checklist and explanation. Ann Intern Med.

[28] Hennink MM, Kaiser BN, Marconi VC (2017). Code saturation versus meaning saturation: how many interviews are enough?. Qual Health Res.

[29] Saunders B, Sim J, Kingstone T, Baker S, Waterfield J, Bartlam B, Burroughs H, Jinks C (2018). Saturation in qualitative research: exploring its conceptualization and operationalization. Qual Quant.

[30] Fugard AJ, Potts HW (2015). Supporting thinking on sample sizes for thematic analyses: a quantitative tool. Int J Soc Res Methodol.

[31] Guest G, Bunce A, Johnson L (2006). How many interviews are enough?: an experiment with data saturation and variability. Field Methods.

[32] Loria S (2020). TextBlob: Simplified Text Processing - Release v0.16.0. TextBlob.

[33] Pang B, Lee L (2008). Opinion mining and sentiment analysis. Found Trends Inf Retr.

[34] Anderson NH (1968). Likableness ratings of 555 personality-trait words. J Pers Soc Psychol.

[35] Tausczik YR, Pennebaker JW (2009). The psychological meaning of words: LIWC and computerized text analysis methods. J Lang Soc Psychol.

[36] De Choudhury M, Gamon M, Counts S, Horvitz E (2021). Predicting depression via social media. Proc Int AAI Conf Web Soc Media.

[37] Park M, Cha C, Cha M (2012). Depressive moods of users portrayed in Twitter. Proceedings of the 18th ACM International Conference on Knowledge Discovery and Data Mining.

[38] Du J, Xu J, Song H, Liu X, Tao C (2017). Optimization on machine learning based approaches for sentiment analysis on HPV vaccines related tweets. J Biomed Semantics.

[39] Jilka S, Odoi CM, van Bilsen J, Morris D, Erturk S, Cummins N, Cella M, Wykes T (2022). Identifying schizophrenia stigma on Twitter: a proof of principle model using service user supervised machine learning. Schizophrenia (Heidelb).

[40] Shapiro SS, Wilk MB (1965). An analysis of variance test for normality (complete samples). Biometrika.

[41] Kruskal WH, Wallis WA (1952). Use of ranks in one-criterion variance analysis. J Am Stat Assoc.

[42] Fay MP, Proschan MA (2010). Wilcoxon-Mann-Whitney or t-test? On assumptions for hypothesis tests and multiple interpretations of decision rules. Stat Surv.

[43] Wilcoxon F (1945). Individual comparisons by ranking methods. Biometrics Bull.

[44] Christensen RH (2019). A Tutorial on fitting Cumulative Link Mixed Models with clmm2 from the ordinal Package. CRAN R Project.

[45] Bolker B, Mächler M, Bates D, Scheipl F, Sørensen Ø, Green P, Chirico M, Alday P, Walker S, Padgham M, Ogden H, Wickham H, Kennedy D, Fultz N, Eddelbuettel D, Henry L, Fellows I, Campbell B, Tappe K, Harris DJ, Singmann H, Armstrong D, Hammill C, Ooms J, Kretch D, Rocks B, Pritikin J, McCloy D (2020). lme4: Mixed-effects models in R. CRAN R Project.

[46] R Core Team (2022). The R Stats Package. Version 4.3.0. CRAN R Project.

[47] Fisher RA (1935). The logic of inductive inference. J Royal Stat Soc.

[48] Benjamini Y, Hochberg Y (1995). Controlling the false discovery rate: a practical and powerful approach to multiple testing. J R Stat Soc Series B Stat Methodol.

[49] R Core Team (2021). R: a language and environment for statistical computing. R Foundation for Statistical Computing.

[50] Fuller-Tyszkiewicz M, Richardson B, Klein B, Skouteris H, Christensen H, Austin D, Castle D, Mihalopoulos C, O'Donnell R, Arulkadacham L, Shatte A, Ware A (2018). A mobile app-based intervention for depression: end-user and expert usability testing study. JMIR Ment Health.

[51] Matthews M, Voida S, Abdullah S, Doherty G, Choudhury T, Im S, Gay G (2015). In situ design for mental illness: considering the pathology of bipolar disorder in mHealth design. Proceedings of the 17th International Conference on Human-Computer Interaction with Mobile Devices and Services.

[52] Matthews M, Abdullah S, Murnane E, Voida S, Choudhury T, Gay G, Frank E (2016). Development and evaluation of a smartphone-based measure of social rhythms for bipolar disorder. Assessment.

[53] Rohani DA, Tuxen N, Lopategui AQ, Faurholt-Jepsen M, Kessing LV, Bardram JE (2019). Personalizing mental health: a feasibility study of a mobile behavioral activation tool for depressed patients. Proceedings of the 13th EAI International Conference on Pervasive Computing Technologies for Healthcare.

[54] Bardram JE, Frost M, Szántó K, Faurholt-Jepsen M, Vinberg M, Kessing LV (2013). Designing mobile health technology for bipolar disorder: a field trial of the monarca system. Proceedings of the SIGCHI Conference on Human Factors in Computing Systems.

[55] Saunders KE, Bilderbeck AC, Panchal P, Atkinson LZ, Geddes JR, Goodwin GM (2017). Experiences of remote mood and activity monitoring in bipolar disorder: a qualitative study. Eur Psychiatry.

[56] McClelland GT, Fitzgerald M (2018). A participatory mobile application (app) development project with mental health service users and clinicians. Health Educ J.

[57] Smith B, Magnani JW (2019). New technologies, new disparities: the intersection of electronic health and digital health literacy. Int J Cardiol.

[58] Steinhubl SR, Muse ED, Topol EJ (2013). Can mobile health technologies transform health care?. JAMA.

[59] Neuhauser L (2017). Integrating participatory design and health literacy to improve research and interventions. Inf Serv Use.

[60] Vayena E, Haeusermann T, Adjekum A, Blasimme A (2018). Digital health: meeting the ethical and policy challenges. Swiss Med Wkly.

[61] Wind TR, Rijkeboer M, Andersson G, Riper H (2020). The COVID-19 pandemic: the 'black swan' for mental health care and a turning point for e-health. Internet Interv.

[62] Torous J, Jän Myrick K, Rauseo-Ricupero N, Firth J (2020). Digital mental health and COVID-19: using technology today to accelerate the curve on access and quality tomorrow. JMIR Ment Health.

[63] Yarrington JS, Lasser J, Garcia D, Vargas JH, Couto DD, Marafon T, Craske MG, Niles AN (2021). Impact of the COVID-19 pandemic on mental health among 157,213 Americans. J Affect Disord.

[64] Strudwick G, Sockalingam S, Kassam I, Sequeira L, Bonato S, Youssef A, Mehta R, Green N, Agic B, Soklaridis S, Impey D, Wiljer D, Crawford A (2021). Digital interventions to support population mental health in Canada during the COVID-19 pandemic: rapid review. JMIR Ment Health.

[65] Turchioe MR, Myers A, Isaac S, Baik D, Grossman LV, Ancker JS, Creber RM (2019). A systematic review of patient-facing visualizations of personal health data. Appl Clin Inform.

[66] RADAR-CNS: Remote Assessment of Disease and Relapse – Central Nervous System. RADAR-CNS.

[67] IMI Innovative Medicines Initiative.

