# Supplementary material for: Health Tracking via Mobile Apps for Depression Self-management: Qualitative Content Analysis of User Reviews
Source: JMIR Hum Factors. 2022 Nov 23;9(4):e40133. doi: 10.2196/40133 (PMC9730209; doi:10.2196/40133)
Supplement: Multimedia Appendix 1 [file humanfactors_v9i4e40133_app1.docx]

## Multimedia Appendix 1 to

# Health tracking via mobile apps for depression self-management: a qualitative content analysis of user reviews

| **Supplementary Figures** | **Location** |
| --- | --- |
| **Figure S1: Flow chart describing app and review screening** | *p. 2* |
| **Figure S2:** **Saturated codes applied to user reviews from Depression self-management apps** | *p. 3* |
|  |  |
| ***Supplementary Tables*** |  |
| **Table S1: Characteristics of included apps** | *provided in Multimedia Supplement 2 (*.xlsx file)* |
| **Table S2: Changes in ratings and sentiment scores over time** | *provided in Multimedia Supplement 2 (*.xlsx file)* |
| **Table S3: Sentiment scores and user ratings associated with review content** | *p. 4* |
| **Table S4: Themes, underlying codes, and illustrative quotes comprising the theme “Designing impactful health tracking mechanisms.”** | *p. 5* |
| **Table S5: Themes, underlying codes, and illustrative quotes comprising the theme “Implementing impactful health tracking mechanisms.”** | *p. 8* |


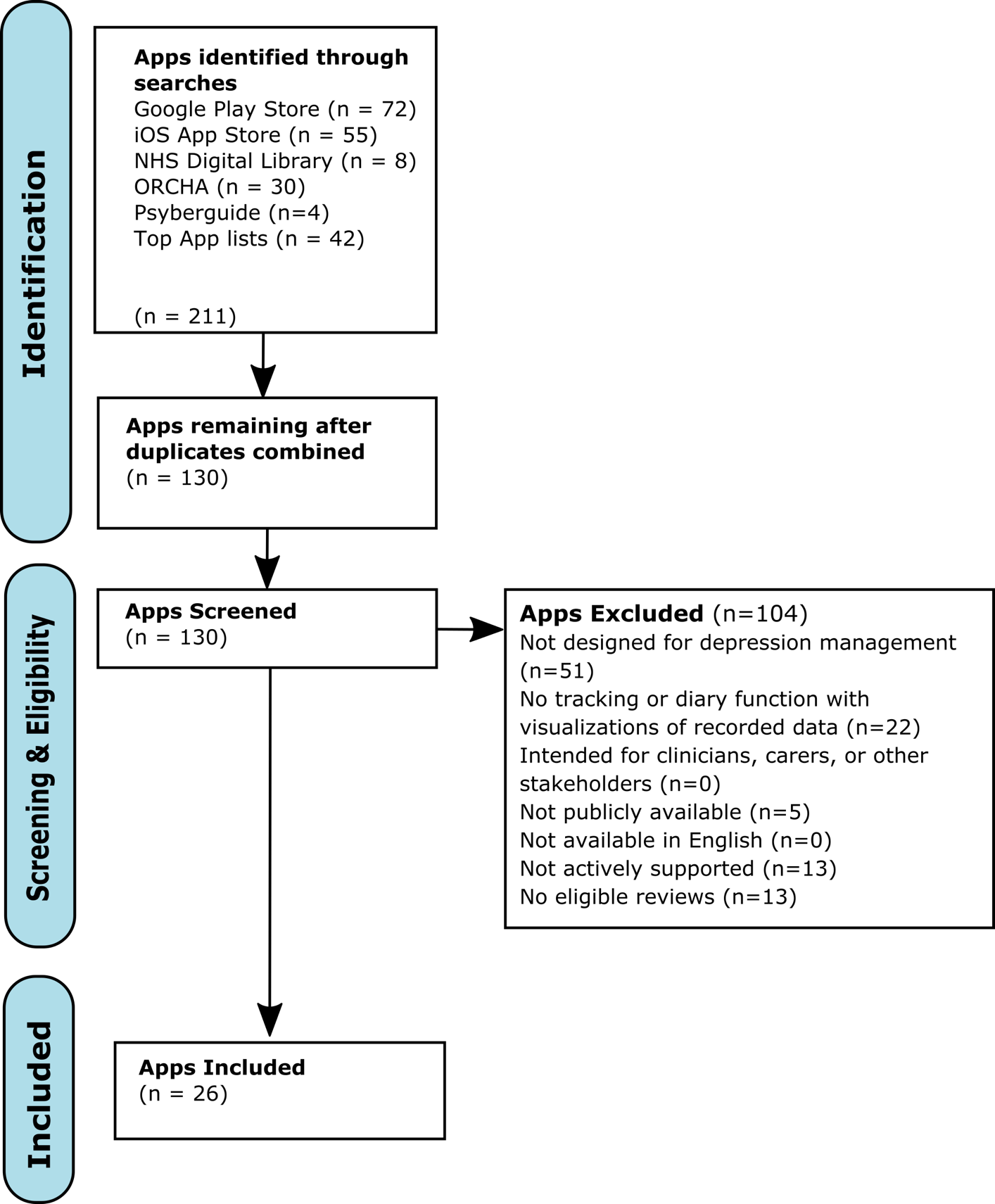


**Figure S1: Flow chart describing app and review screening.** Summed totals of excluded apps exceed the number of screened apps because apps often met multiple exclusion criteria.

**Figure S2:** **Saturated codes applied to user reviews from depression self-management apps.** The four panels describe A) the apps and the number of reviews per app included in this review, B) the number of codes which were identified in the reviews of each app, C) the number of reviews which contained each code, and D) the number of reviews which contained codes related to each descriptive theme. Codes which did not reach saturation, defined as identified in at least five reviews, are not shown.


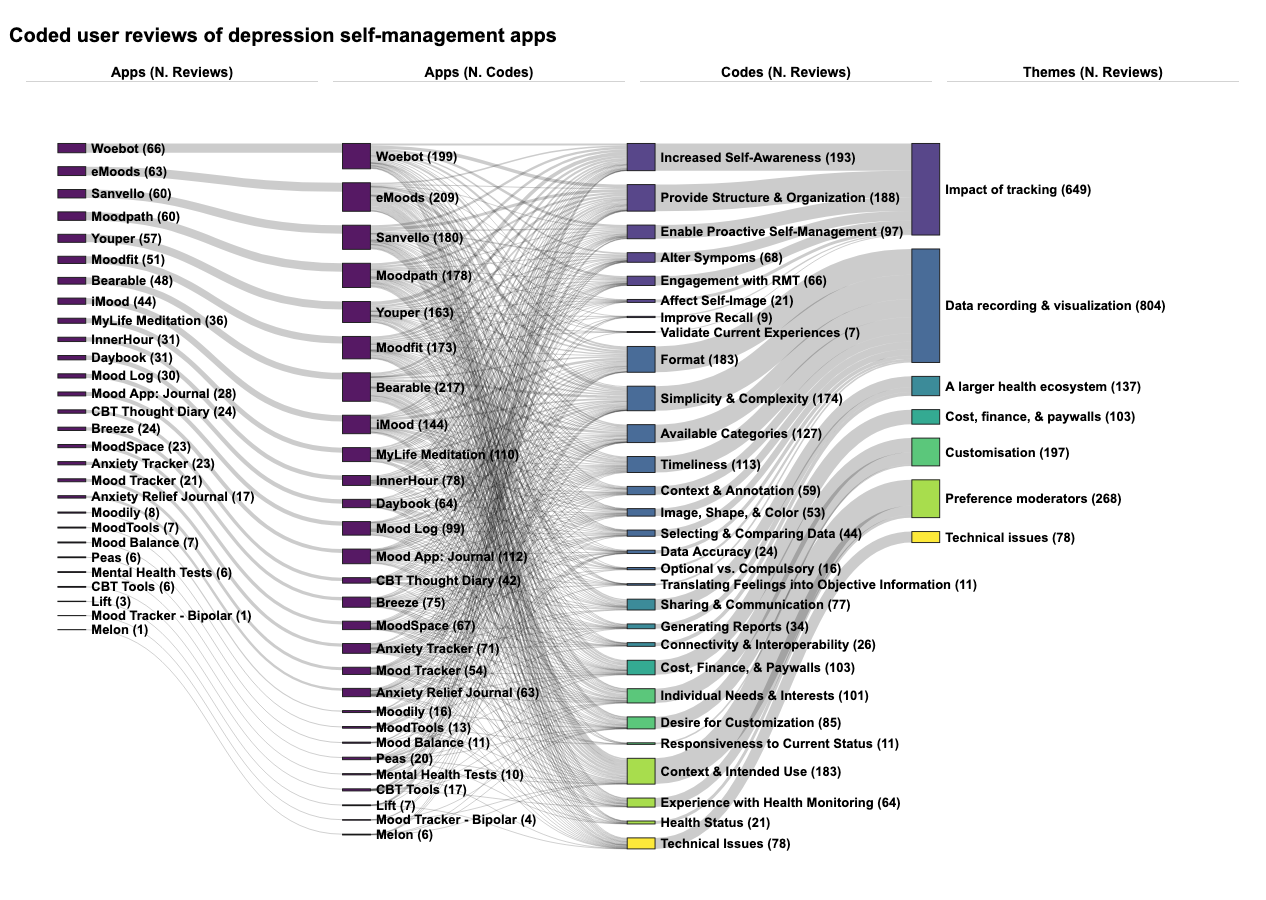


**Table S1: Sentiment scores and user ratings associated with review content**

| **Code** | **N** | **Rating**  Median [Q2-Q3] (p) | **Sentiment Score**  Median [Q2-Q3] (p) |
| --- | --- | --- | --- |
| ***Impact of app-based health tracking*** | | | |
| Increased self-awareness | 193 | 5 [5 - 5] (0.002) ^b^ | 0.27 [0.14 - 0.38] (0.914) |
| Provide structure & organization ^a^ | 188 | 5 [5 - 5] (0.000) ^b^ | 0.25 [0.15 - 0.38] (0.827) |
| Enable proactive self-management | 97 | 5 [5 - 5] (0.001) ^b^ | 0.25 [0.14 - 0.37] (0.684) |
| Altering symptoms | 68 | 5 [5 - 5] (0.045) ^b^ | 0.24 [0.12 - 0.31] (0.114) |
| Engagement with apps | 66 | 5 [5 - 5] (<0.001) ^b^ | 0.26 [0.19 - 0.38] (0.809) |
| Affecting self-image ^a^ | 21 | 5 [5 - 5] (0.323) | 0.22 [0.13 - 0.28] (0.309) |
| Improve recall of past experiences | 9 | 5 [4 - 5] (0.844) | 0.23 [0.18 - 0.30] (0.684) |
| Validate current experiences | 7 | 5 [5 - 5] (0.514) | 0.26 [0.20 - 0.32] (0.827) |
| ***Designing impactful health tracking apps*** | | | |
| *Recording, reviewing, visualizing data* |  |  |  |
| Format | 183 | 5 [4 - 5] (0.058) | 0.25 [0.14 - 0.36] (0.267) |
| Simplicity & complexity | 174 | 5 [5 - 5] (0.045) ^b^ | 0.30 [0.19 - 0.41] (0.028) ^b^ |
| Available categories ^a^ | 127 | 5 [4 - 5] (0.008) ^b^ | 0.25 [0.14 - 0.38] (0.682) |
| Timeframe | 113 | 5 [4 - 5] (0.827) | 0.24 [0.13 - 0.35] (0.261) |
| Context & annotation ^a^ | 59 | 5 [4.5 - 5] (0.399) | 0.24 [0.13 - 0.36] (0.598) |
| Image, shape, & color | 53 | 5 [4 - 5] (0.060) | 0.21 [0.12 - 0.35] (0.261) |
| Selecting & comparing data streams | 44 | 4 [4 - 5] (<0.001)^b^ | 0.16 [0.10 - 0.29] (0.003) ^b^ |
| Optional vs compulsory | 16 | 5 [4 - 5] (0.588) | 0.21 [0.13 - 0.29] (0.267) |
| Translating feelings into objective information | 11 | 5 [4.5 - 5] (0.974) | 0.27 [0.14 - 0.40] (0.947) |
| *Customization* |  |  |  |
| Individual needs & interests ^a^ | 101 | 5 [4 - 5] (0.784) | 0.29 [0.19 - 0.40] (0.332) |
| Desire for customization | 85 | 5 [4 - 5] (0.177) | 0.29 [0.17 - 0.39] (0.684) |
| Responsiveness to current status | 11 | 4 [4 - 5] (0.054) | 0.17 [0.12 - 0.21] (0.171) |
| *Preference moderators* |  |  |  |
| Context & intended use ^a^ | 183 | 5 [5 - 5] (0.106) | 0.25 [0.16 - 0.37] (0.684) |
| Experience with health monitoring | 64 | 5 [5 - 5] (<0.001) ^b^ | 0.26 [0.17 - 0.35] (0.827) |
| Health status | 21 | 4 [4 - 5] (0.058) | 0.17 [0.12 - 0.23] (0.014) ^b^ |
| ***Implementing impactful health tracking apps*** | | | |
| *A larger health ecosystem* |  |  |  |
| Sharing & communication ^a^ | 77 | 5 [5 - 5] (0.058) | 0.24 [0.17 - 0.38] (0.914) |
| Generating & exporting reports ^a^ | 34 | 5 [4 - 5] (0.236) | 0.25 [0.11 - 0.41] (0.914) |
| Connectivity & interoperability | 26 | 4 [4 - 5] (0.001) ^b^ | 0.22 [0.16 - 0.36] (0.736) |
| *Cost, finance and paywalls* |  |  |  |
| Cost, finance and paywalls | 103 | 5 [4 - 5] (0.162) | 0.25 [0.15 - 0.36] (0.261) |
| ***Technical issues: Data loss and inaccuracies*** |  |  |  |
| Technical issues ^a^ | 78 | 4 [3 - 4] (<0.001) ^b^ | 0.14 [0.04 - 0.29] (<0.001) ^b^ |
| Accuracy of data | 24 | 4 [3.75 - 4.25] (<0.001) ^b^ | 0.25 [0.05 - 0.35] (0.267) |
| N reflects the number of reviews for which the code was used.  p values reflect Benjamini-Hochberg correction.  ^a^ Relative frequency of the code changed over time, determined through fisher exact tests on the proportion of reviews using the code per year  ^b^ Ratings or sentiment scores differed from the rest of the review corpus, determined through Wilcoxon signed-rank tests | | | |

**Table S2: Themes, underlying codes, and illustrative quotes comprising the theme “Designing impactful health tracking mechanisms.”** Column one describes the number of times each code was identified (N) and the whether the code was deductive or inductive. Column two describes code definitions and illustrative quotes (provided in italics).

| **Codes** | **Code definition & illustrative quotes** |
| --- | --- |
| ***Recording, reviewing, and visualizing data*** | |
| **Format**  N=183, Deductive | Description of the form of the health tracking mechanism.  *"I’ve been using this app for 4 years. This app allows you to produce a diary of your day to day mood/life similar to most apps. You can attach a picture of yourself or anything. You can add any number of entries daily. There’s also the hashtag system which is brilliant. It allows to track an unlimited number of factors that could be affecting your mood..." (iMood, 2021, 5 stars)*  *"Overall, this application is good. But, in diary, it would be nice if you can change date input with calendar pick (like monthly calendar). Current date input (if we prefer to change date ) does not inform what day it was (Sunday, or Monday etc). That would be a great help because I often remember what day it was. Not date." (Mood Tracker, 2020, 2 stars)*  *"Good so far could do with a little more functionality to the graph such as adding a separate line for medication or alcohol consumption, excercise etc." (Mood Log, 2021, 4 stars)*  *"Zero stars. This is nothing more than a journal app with some very basic emojis meant to represent complex emotions, and is completely useless for managing real anxiety." (Anxiety Relief Journal, 2020, 1 star)* |
| **Simplicity & complexity**  N=174, Deductive | Any discussion regarding service users’ preferences on how simple or complex a data reporting mechanism or visualization should ideally be. This code included preferences, general comments, and tensions relating to simplicity and complexity.  *"Love seeing a graph of my mood and SO pleased I can customize my mood colours. Not complicated but still has all the features I want. Very happy!" (Mood Log, 2021, 5 stars)*  *"Was good once. Developers have over-complicated what should be a simple calm process. I need only 1-2 time tested meditations (e.g. breathing) delivered in the same way every time. (Variety is not the point of meditation! ). Now I find no easy one click way to get to my favourite meditation and no escape from having to 'check in' and track progress. I would also like an offline version as I cannot use it on many planes or rural settings." (MyLife Meditation, 2018, 2 stars)* |
| **Available categories**  N=127, Inductive | Any discussion of the types of data, moods, or detailed options service users wished to track or visualize in an app  *"Since going through the entire list of words l—letters A through Z—is tedious, I’m too frustrated with all back-and-forth to even keep review anything after the letter C. Surely, you want to help people gather a representative account of their moods. Even if you have, say, a 10-word cutoff to endure some self-reflection to make the list as accurate as possible. Without easy way to select from a variety of words, i feel I’m missing a true sense of day-to-day distinction around my feelings as I’m tracking them… And the automatic multiple choice list of adjectives does take the guesswork out of tracking your feelings. But again, for those of us who are curious and want to put extra thought and time into browsing and selecting from a broader range of descriptions, don’t make it harder and ultimately discouraging to do so. " (Moodpath, 2020, 4 stars)* |
| **Timeliness**  N=113, Deductive | Any description of how often, how quickly, or over what time period individuals preferred to track, access and visualize their data.  *"Updating my review after bug fixes, love this app, very helpful. Is there anyway to set up multiple notifications in a day, i would love to track my anxiety at different times of day." (Anxiety Tracker, 2020, 5 stars)*  *"hi I really like this mood tracker it would just be nice if you could see more than just today. Is there away to show this maybe I'm missing it great app though" (Peas, 2019, 3 stars)* |
| **Context & annotation**  N=59, Deductive | Any discussion related to contextual information required to interpret and use the visualization. Context could be internal (i.e., self-reported notes to explain a specific score), or external (information provided by the app to help users interpret and act on the visualized data).  *"I love everything about this app. It lets you create posts with so many elements. A little journaling, add a picture, a location tag, even hashtags that are grouped into positive and negative. They also offer a wide range of moods to pick from when you start the entry. It tracks trends and shows overall patterns which is super helpful for tracking my moods." (iMood, 2021, 5 stars)* |
| **Image, shape, & color**  N=53, Deductive | Any discussion regarding ways in which image, shape, and color affected users’ experiences or preferences of a visualization.  *"The best. I installed a whole bunch of mood trackers and this is the one I am keeping. Can view as a mood graph, colour coded calendar, sort and file based on tags (like medications, emotions, physical symptoms - all completely customisable. And can set up to 3 reminders a day at any time, perfect for getting an accurate picture." (Mood Log, 2018, 5 stars)* |
| **Selecting & comparing data streams**  N=44, Deductive | Any discussion of selecting (e.g., choosing which data to visualize), manipulating (e.g., zooming in, analyzing or filtering), or comparing data streams that app reviewers were tracking  *"Personally I’d prefer the monthly/ yearly overview graph had depressed averages below the elevated averages. This would more clearly give a graph like presentation with higher periods being at the top of the graph and lower periods lower in the graph. I wonder if this could be added as a customisable setting in the ‘log moods’ and ‘graph’ settings." (eMoods, 2020, 5 stars)*  *"The data screen is not that useful as it lumps all the medication together, so although you can chart it tracked against mood you can’t tell what meds correlate to what changes." (Moodfit, 2019, 4 stars)* |
| **Optional vs compulsory**  N=16, Inductive | Any discussion of whether data entry and tracking should be optional or required in an app  *"...Also, let’s talk about MoodPath Guilt. It’s Easy to feel guilty because you’re not doing the tracking three times a day. there should be an option to start with tracking just once a day and still receiving a result. Maybe there are levels of engagement—but please don’t add a pay wall or premium— where you graduate from once a day Moodpath-ing after 30 days when you move to twice a day. The idea is you working your way up to three times a day after three months. But! Maybe there’s “go back to 1xday” or “go back to 2xday” option that the app presents if/when the user misses a check in. Then you can continue working your way toward the three times daily goal. This way, Moodpath becomes a habit-building instrument that fosters self-esteem which is a bonus positive effect beyond just collecting mood data." (Moodpath, 2020, 4 stars)* |
| **Translating feelings into objective information**  N=11, Deductive | Any discussion of using data reporting mechanisms and visualizations to translate abstract experiences, feelings, or symptoms into objective data  *"… I am so thankful that I have found an outlet to express myself and monitor my mental health in a safe environment…” (Sanvello, 2018, 5 stars)*  *"I really like this app, it helps me keep track of my day to day life and my thoughts and actions. It would be nice if it had a drawing like feature added to it so I can doodle out my ideas and thoughts when I cant describe them." (Daybook, 2020, 5 stars)* |
| ***Customization*** | |
| **Individual needs & interests**  N=101, Deductive | Any discussion of customization, flexibility, personalization, or conflicting opinions due to individual experiences, priorities, and preferences  *"I have bi polar disorder, and one of the most important tools in treatment aside from medication and talk therapy is tracking mood and sleep etc. Bearable is the best app out there for this purpose - it has exactly everything you need, and if it doesn’t then it is easily customisable to add extra moods or symptoms in. Once customised, you can easily tap on a symptom and even rate its severity." (Bearable, 2020, 5 stars)* |
| **Desire for customization**  N=85, Deductive | Indication of a desire (or lack thereof) for customizing health tracking features  *"this is really the best mood tracker...but i hope you can add a feature which we can change the colors of the trackers and more themes.. and that really all of my suggestion i mean the app is already perfect" (Bearable, 220, 5 stars)* |
| **Responsiveness to current status**  N=11, Deductive | Any discussion of customization, flexibility, personalization, or conflicting opinions due to individual experiences, priorities, and preferences  *"I love that it tracks your mood and that you can add custom emotions to better express yourself. I used this with my psychiatrist, helped with tracking how I was doing during medication switches and the little journal entry allows you to be more detailed. Even if you don’t feel like adding a full entry, the app gives you a few questions to put in simple entries so you aren’t missing a bunch (we all know how daunting tasks feel when your feeling low)." (Moodpath, 2019, 4 stars)*  *"Helpful for me when I was struggling w anxiety disorder. Still use. I like the gratitude journal as well but this app has so many adjustable features" (Moodfit, 2020, 5 stars)* |
| ***Preference moderators*** | |
| **Context & intended use**  N=183, Deductive | Any discussion of how context, including personal goals and intentions, impacts (actually or hypothetically, implicit or explicit) current design preferences  *"I don’t have bipolar disorder, but I needed a mood tracker to keep track of my depression. I tried Mood tracker after mood tracker, and I kind of just hated all of them. All I wanted was a simple, easy to use app that made keeping track of depression easy; I didn’t need: emojis, a social media component, bright cheerful colors/positive quotes. I finally found this one when scrolling through the App Store one day. The scale from mild to moderate for emotions makes it easier to decide how to label my mood than a ton of verbs like “good”, “bad”, “meh”, etc. it also made it easy to keep track of my sleep, and write notes about how I’m feeling. Would I recommend it? Yes, if you want a simple no frills app that takes tracking moods seriously try it out." (eMoods, 2018, 5 stars)*  *"I’m sure it’s a great app for some people which is why I gave it 3 stars. But it doesn’t work at all for my needs so now I’m out $4. :( I wish Apple store offerred refunds. Site says “track anything you like, sleep, anxiety, energy levels, etc”. But it doesn’t say you can only track 1 thing! I need to track mood and energy level and irritability separately. And now I’m unhappy and irritated that I can’t." (iMood, 2018, 2 stars)* |
| **Experience with health monitoring**  N=64, Deductive | Any discussion of how past experiences with health tracking or mHealth applications impact (actually or hypothetically, implicit or explicit) current design preferences  *"I’ve tried a bunch mood trackers but deleted all of them. This is only one that works for me. I really enjoy the layout and the level of customization. The key thing is the customization allows you to see via graphs any patterns (“insights” in app). It’s easy to track as much or as little as you want." (Bearable, 2020, 5 stars)*  *"Was looking for a way to track my bipolar 1 moods and looked at a few apps. eMoods stood out as the easiest one to meet my needs. Moods, meds, journal, sleep, etc., and with a $.99 upgrade I can customize it to suit my daily needs. Great tool!" (eMoods, 2019, 5 stars)* |
| **Health status**  N=21, Deductive | Any description of how health status impacts (actually or hypothetically, implicit or explicit) design preferences. This could include impact of a user’s condition or disease severity (i.e., preferences in moderate vs. severe disease), differing preferences before/during/after relapses or episodic events, or preferences in times of wellness vs. those while feeling unwell.  *"This is the best app I’ve ever used to track my moods, set goals for myself to stay or become mentally healthy, and I also use it as a journal sometimes. I can’t believe all of the options of featured this app has yet its not complicated or time consuming. Which is the last thing I need when I’m feeling fatigued, depressed, and/or overwhelmed!!..." (Moodfit, 2018, 5 stars)* |

**Table S3: Themes, underlying codes, and illustrative quotes comprising the theme “Implementing impactful health tracking mechanisms.”** Column one describes the number of times each code was identified (N) and the whether the code was deductive or inductive. Column two describes code definitions and illustrative quotes (provided in italics).

| **Codes** | **Code definition & illustrative quotes** |
| --- | --- |
| ***A larger health ecosystem*** | |
| **Sharing & communication**  N=77, Deductive | Any discussion of real or hypothetical use of app-based tracking mechanisms to communicate with others, including the rationale, experiences, preferences, and outcomes of using visualizations in those contexts.  *"This helped me keep track of my feeling and thoughts and gave advice when needed. It also give a letter to a doctor if needed at the end which makes it easyer to seek the help you need." (Moodpath, 2018, 5 stars)*  *"This app is amazing. So helpful in keeping track and trying to explain to your doctor what you are you going through, when, and to what degree. You can add custom symptoms, see charts, etc. . thank you for creating this - it's so helpful and makes things easy even when you are dealing with not feeling well." (Bearable, 2020, 5 stars)* |
| **Generating & exporting reports**  N=34, Inductive | Any description of or requests for features which enabled users to export their data, either in a formatted report or as raw data.  *"Love everything about this app. So easy to set up and use, doesn't overload you with notifications or ads, only takes moments to track items, and the tools are super-useful. I've been using for a week and already it's part of my routine.The only suggestions I have are: * It would really useful to be able to download the Insights - I love that you can see how the different categories impact mood, but being able to see them all together in one chart would be the next step * Graphics could be improved upon All-in-all the best app of this kind; thank you developers! 😁" (Moodfit, 2018, 5 stars)*  *“Has evereything you want. Easy to log and keep track of history. Would however love a Cloud-based back-up solution or a way to export data by e-mail." ()Anxiety Tracker, 2019, 5 stars* |
| **Connectivity & interoperability**  N=26, Inductive | Any description of or requests for features which enabled a self-management app to connect with and import data from other health, wellness, or self-management applications  *"I would have given this app 5 stars but the Google drive backup doesn’t work." (iMood, 2021, 4 stars)*  *"This app really works for me and you can sync across iOS devices." (Bearable, 2020, 5 stars)*  *"I hope this app will soon work with Apple health, Fitbit app and Loseit! (which is by far the most easy to use and more comprehensive food tracker)." (eMoods, 2018, 4 stars)* |
| ***Cost, finance, or paywalls*** | |
| **Cost, finance, or paywalls**  N=103, Inductive | Any discussion of paid access to the app or app features.  *"I have bipolar II & adhd. Regulating my emotions and organizing my thoughts can be extremely difficult at times. This app literally feels like a miracle. 2 days into my 7 day free trial of the membership, I was sold. I can track my moods and sleep, check off daily goals (and set reminders to do them!), see trends over time and see how they correlate with other variables, record thought distortions and challenge them, ground myself back from a panic attack, practice daily gratitude, and more. All that for $60 a year, that’s not bad at all. …. Highly highly recommend this app. & it’s 1000% worth the paid subscription. Hey Ron, I only have like 150 followers on social media but can I advertise for you? 😂 Not hard when you believe in the product" (Moodfit, 2020, 5 stars)*  *"Very helpful for tracking your mood and helping you feel better. It takes you into your thoughts to realize why you're feeling how you do and to help you cope. It is very organized in a helpful way with a simple graph. But, ever since the new update, the app has been very slow, and has locked a majority of the content behind a paywall." (Youper, 2021, 4 stars)*  *"I just wanted to get a free app to track my mood and whatever. But instead it wants me to pay at least £7.99 a week!! Terrible:(" (Mood Balance, 2021, 1 star)* |
| ***Technical issues: Data access and accuracy*** | |
| **Technical issues**  N=78, Inductive | Any description of technical issues which involved or impeded a user’s ability to track or visualize their data.  *"I had been using this app for almost a year when it suddenly wiped out my 349 day streak and completely lost all my information!! I can’t remember the password, I’m completely frustrated with this app! It was good, but now it is useless" (MyLife Meditation, 2021, 1 star)*  *"I've been using this app for about 2 months or so now and I really like it. However , there is some kind of bug happening right now; the app is not uploading new pictures for my entries, it replaced the selfie I took with a shot from 4 or 5 days ago. I've had the same picture log for the last 4-5 days, and that aspect is one of the reasons I love it so much. Now it seems to be useless. I've tried contacting for support via the website with no reply. Thanks!" (iMood, 2020, 4 stars)* |
| **Accuracy of data**  N=24, Deductive | Any discussion of how a service user perceives the accuracy of a data tracking mechanism  *"I just tracked my mood today, November 20th, and it’s tracking it as November 21st. It does this all the time. It tracks/counts days wrong, which makes it impossible to get an accurate idea of what happened when and how it affected you that day. I want to like this app, but this is ridiculous." (Breeze, 2019, 2 stars)* |
